# *Distoseptispora bambusae* sp. nov. (Distoseptisporaceae) on bamboo from China and Thailand

**DOI:** 10.3897/BDJ.8.e53678

**Published:** 2020-06-01

**Authors:** Yaru Sun, Ishani D. Goonasekara, Kasun M. Thambugala, Ruvishika S. Jayawardena, Yong Wang, Kevin D. Hyde

**Affiliations:** 1 Department of Plant Pathology, College of Agriculture, Guizhou University, Guiyang, China Department of Plant Pathology, College of Agriculture, Guizhou University Guiyang China; 2 Center of Excellence in Fungal Research, Mae Fah Luang University, Chiang Rai, Thailand Center of Excellence in Fungal Research, Mae Fah Luang University Chiang Rai Thailand; 3 Genetics and Molecular Biology Unit, Faculty of Applied Sciences, University of Sri Jayewardenepura, Gangodawila, Nugegoda, Sri Lanka Genetics and Molecular Biology Unit, Faculty of Applied Sciences, University of Sri Jayewardenepura Gangodawila, Nugegoda Sri Lanka; 4 Institute of Plant Health, Zhongkai University of Agriculture and Engineering, Haizhu District, Guangzhou, China Institute of Plant Health, Zhongkai University of Agriculture and Engineering, Haizhu District Guangzhou China

**Keywords:** One new taxon, Distoseptisporales, hyphomycete, multi-gene phylogeny, taxonomy

## Abstract

**Background:**

Bamboo is a widespread plant with medicinal value. During our taxonomic study on medicinal plants, three collections of *Distoseptispora* were made from China and Thailand. Phylogenetic analyses of combined LSU, ITS and *RPB2* sequence data showed that two collections represented a new species, phylogenetically distinct from other described species in *Distoseptispora*.

**New information:**

This new species has macronematous, mononematous conidiophores, polyblastic or monoblastic conidiogenous cells and acrogenous, solitary, straight, obclavate, multi-septate, thick-walled conidia. *Distoseptispora bambusae* sp. nov. is introduced with illustrations and a comprehensive description. The third collection on dead wood from Thailand was identified as *D. tectona* with newly-generated molecular data for this taxon.

## Introduction

*Distoseptispora* was introduced by Su et al. (2016) with *Distoseptispora fluminicola* McKenzie, H.Y. Su, Z.L. Luo & K.D. Hyde as the type species. *Distoseptispora* has macronematous, septate, unbranched, straight or flexuous, smooth, olivaceous to brown conidiophores; mono- or polyblastic, holoblastic, determinate, terminal, cylindrical conidiogenous cells and acrogenous, solitary, olivaceous to brown, euseptate or distoseptate conidia (Su et al. 2016, Luo et al. 2018, Yang et al. 2018, Hyde et al. 2019, Luo et al. 2019). The monotypic family Distoseptisporaceae was established to accommodate *Distoseptispora* in Sordariomycetes (Su et al. 2016, Hyde et al. 2020). The freshwater genus *Aquapteridospora* J. Yang, K.D. Hyde & Maharachch was introduced by Yang et al. (2015) and was treated as Diaporthomycetidae genera *incertae sedis*, based on LSU sequence data. In a comprehensive study of freshwater Sordariomycetes, Luo et al. (2019) established Distoseptisporales and placed Distoseptisporaceae and *Aquapteridospora* within this order. *Aquapteridospora* differs from *Distoseptispora* in having polyblastic conidiogenous cells, bearing tiny, circular scars and protuberant, fusiform conidia. This treatment was followed by Hyde et al. (2020). However, Wijayawardene et al. (2020) only accepted Distoseptisporaceae in Distoseptisporales, while *Aquapteridospora* was placed in Diaporthomycetidae genera *incertae sedis*.

Currently, 25 species are accepted in *Distoseptospora*, of which 16 are from freshwater habitats and nine from terrestrial (Luo et al. 2018, Tibpromma et al. 2018, Crous et al. 2019, Hyde et al. 2019, Luo et al. 2019, Phookamsak et al. 2019). *Distoseptispora caricis* is the only reported endophytic species, while the others are saprobes.

During ongoing surveys of microfungi on medicinal plants, two *Distoseptispora* species were collected in China and Thailand. We introduce *Distoseptispora bambusae* as a novel taxon with illustrations and molecular phylogenetic data. We also provide newly-generated molecular data of the second species, *D. tectona* Doilom & K.D. Hyde, which was also reported from Thailand (Hyde et al. 2016).

## Materials and methods

### Collections and examination of specimens

Specimens of bamboo culms were collected from Guiyang, Guizhou Province, China (August 2019) and Doi Mae Salong, Chiang Rai, Thailand (July 2015). Another specimen of dead wood was collected from the Botanical Garden, Mae Fah Luang University, Chiang Rai, Thailand (November 2019). The samples were processed and examined following the method described by Dai et al. (2017). Samples were brought to the laboratory in an envelope after recording the collection details including hosts, places and dates. Morphological observations were made using a stereomicroscope (SteREO Discovery. V12, Carl Zeiss Microscopy GmBH, Germany). Fruiting bodies were transferred with a needle and placed in a drop of distilled water on a glass slide, then covered with the cover slip for microscopic studies and photomicrography. The morphological figures were captured using a Nikon ECLIPSE Ni compound microscope (Nikon, Japan) fitted with a NikonDS-Ri2 digital camera (Nikon, Japan). Measurements were made using the Tarosoft (R) Image Frame Work software. Photo-plates were made with Adobe Photoshop CS6 software (Adobe Systems, USA).

Single-spore isolations were done following the method described in (Chomnunti et al. 2014). Germinated spores were transferred to potato dextrose agar (PDA: 39 g/l sterile distilled water, Difco potato dextrose) plates and incubated at room temperature for 4 weeks. Herbarium materials were deposited in the Fungarium of Mae Fah Luang University (MFLU), Chiang Rai, Thailand. Pure cultures were deposited in the Mae Fah Luang University Culture Collection (MFLUCC) and International Collection of Microorganisms from Plants (ICMP). Facesoffungi (FoF) and Index Fungorum numbers were acquired as described in Jayasiri et al. (2015) and Index Fungorum (http://www.indexfungorum.org).

### DNA extraction, PCR amplification and sequencing

Fresh fungal mycelia were scraped with sterilised scalpels. Genomic DNA was extracted using Genomic DNA Extraction Kit (GD2416) following the manufacture’s protocol. PCR amplifications were performed in a 20 μl reaction volume, with 10 μl of 10 × PCR Master Mix, 1 μl of each primer, 1 μl template DNA and 7 μl ddH_2_O. Primers used and PCR thermal cycle programmers are listed in Table 1.

### Phylogenetic analyses

Sequences (Table 2) generated during this study were complemented with sequences from previous studies (Hyde et al. 2016, Su et al. 2016, Luo et al. 2018, Crous et al. 2019, Hyde et al. 2019, Luo et al. 2019), which were downloaded from NCBI GenBank (https://www.ncbi.nlm.nih.gov/genbank/). Alignments for each locus were done in MAFFT v7.212 (Katoh and Standley 2013) and checked visually using AliView (Larsson 2014). The alignments were trimmed using trimAl v 1.2 with gappyout (Capella-Gutiérrez et al. 2009). Three single gene alignments were combined using Sequence Matrix (Vaidya et al. 2011). The final alignment was deposited in TreeBASE (submission ID: http://purl.org/phylo/treebase/phylows/study/TB2:S26081).

The Maximum Likelihood (ML) analysis was performed using IQ-tree (Nguyen et al. 2015, Chernomor et al. 2016). Nucleotide substitution models were selected under the Akaike Information Criterion (AIC) by jModelTest2 (Darriba et al. 2012) on XSEDE in the CIPRES web portal (Miller et al. 2010). For ITS dataset, the GTR+I+G model was selected (-lnL=3364.5406), for LSU, the TIM2+I+G model (-lnL = 959.3999), and for *RPB2*, the GTR+I+G (-lnL= 5111.0788). ML was inferred under partitioned models. Non-parametric bootstrap analysis was implemented with 1000 replicates.

Maximum Parsimony (MP) analysis was carried out with the heuristic search in PAUP v. 4.0b10 (Swofford 2002). All characters were unordered and of equal weight, and gaps were treated as missing data. Maxtrees were unlimited, branches of zero length were collapsed and all multiple, equally-parsimonious trees were saved. Clade stability was assessed using a bootstrap (BT) analysis with 1,000 replicates, each with 10 replicates of random stepwise addition of taxa (Hillis and Bull 1993).

Bayesian Inference (BI) analysis was performed by the Markov Chain Monte Carlo sampling (MCMC) coalescent approach implemented in BEAST v1.8.4 (Drummond et al. 2012), with an uncorrelated lognormal relaxed clock. The Birth-Death Incomplete Sampling speciation model (Stadler 2009) was selected as tree prior. The nucleotide substitution models were the same as above. Markov chains were run for 1,000,000 generations and trees were sampled every 1000th generation. The XML file generated by BEAUti (Drummond et al. 2012) was run using BEAST on XSEDE in the CIPRES web portal (Miller et al. 2010). Tracer v1.6 (Rambaut et al. 2014) was used to check the resulting log file. The first 20% of trees, representing the burn-in phase of the analyses, were discarded and a Maximum Clade Credibility tree was inferred using TreeAnnotator 1.8.4.

Trees were visualised with FigTree v1.4.4 (Rambaut 2009) and the layout was edited using Adobe Illustrator CS6 software (Adobe Systems, USA).

## Taxon treatments

### Distoseptispora bambusae

Y.R. Sun, I.D. Goonasekara, Yong Wang bis & K.D. Hyde sp. nov.

580E5F7E-10E0-5AFA-BDF3-FDA7DAB58F19

557452

#### Materials

- **Type status:** Holotype. **Occurrence:** catalogNumber: MFLU 20–0261; recordedBy: Sun Ya-Ru; **Taxon:** scientificName: Distoseptispora bambusae; class: Sordariomycetes; order: Distoseptisporales; family: Distoseptisporaceae; **Location:** country: China; stateProvince: Guizhou; locality: Guiyang Medicinal Plants Garden; verbatimElevation: 1100 m; **Identification:** identifiedBy: Yaru Sun; dateIdentified: 2019
- **Type status:** Paratype. **Occurrence:** catalogNumber: MFLU 17–1653; recordedBy: Thambugala Kasun M.; **Taxon:** scientificName: Distoseptispora bambusae; class: Sordariomycetes; order: Distoseptisporales; family: Distoseptisporaceae; **Location:** country: Thailand; stateProvince: Chiangrai; locality: Doi Mae Salong; verbatimElevation: 390 m; **Identification:** identifiedBy: Yaru Sun; dateIdentified: 2019

#### Description

**Figure 1.**
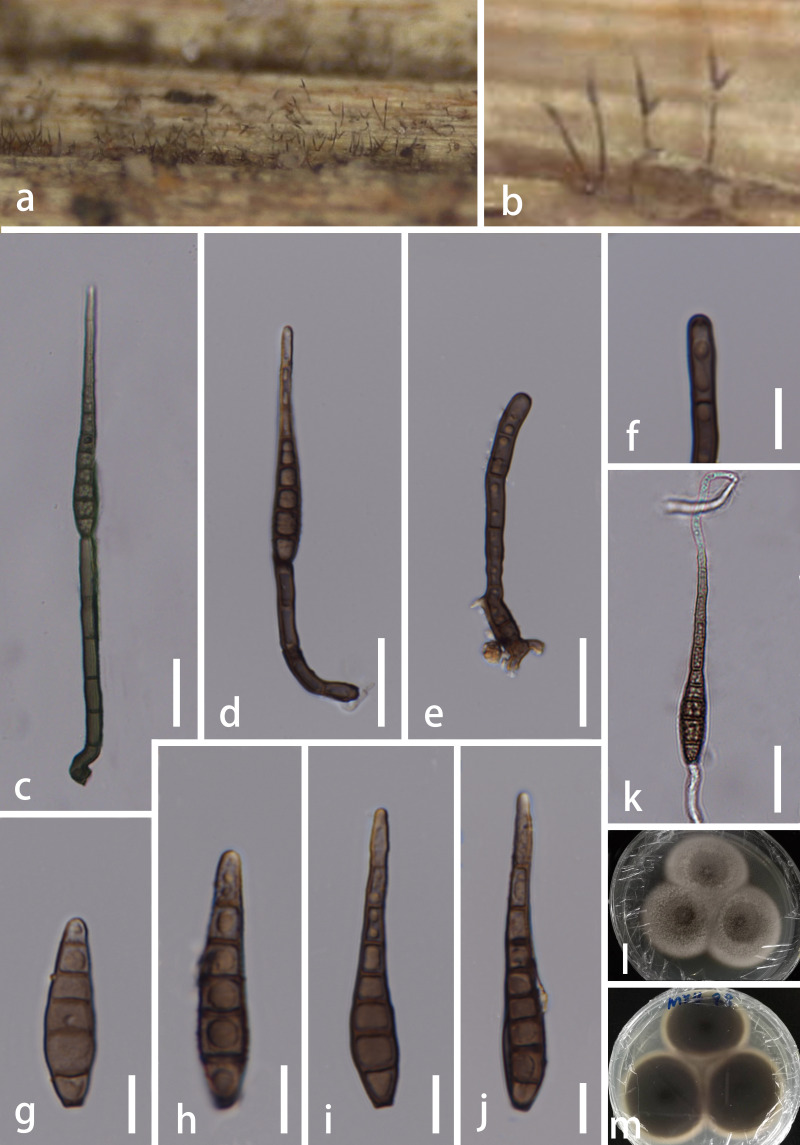
*Distoseptispora bambusae* (MFLU 20–0261, holotype, collected from China) **a, b.** Colonies on natural substrate; **c, d.** Conidiophore with Conidia; **e.** Conidiophore; **f.** Conidiogenous cell; **g**–**j.** Conidia; **k.** Germinating conidium; **l**, **m.** Colony on PDA. Scale bars: c–e, k = 20 μm, f–j = 10 μm.

**Figure 2.**
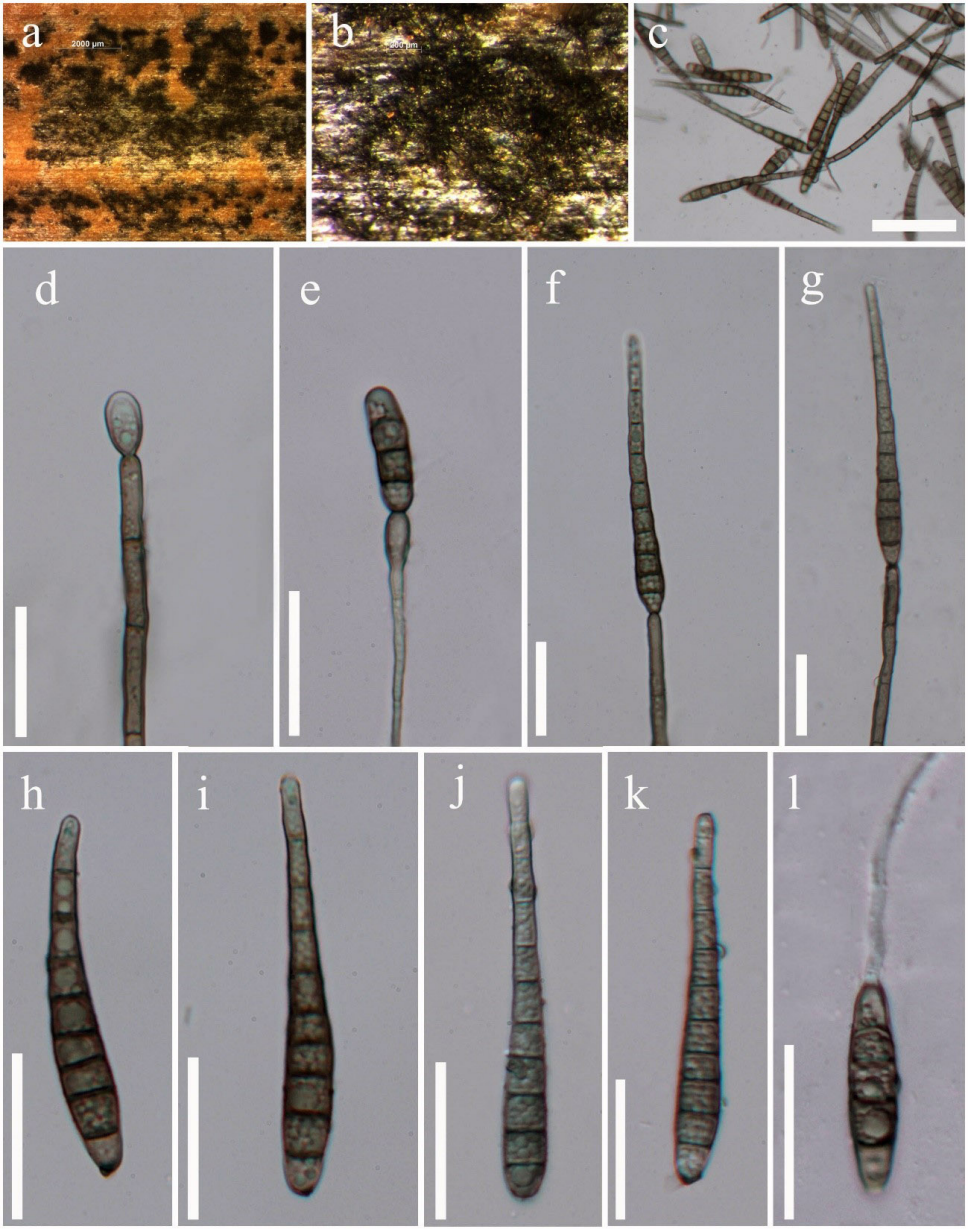
*Distoseptispora bambusae* (MFLU 17–1653, paratype, collected from Thailand). **a, b.** Colonies on natural substrate; **c**–**g.** Conidia attached to conidiophores; **h**–**k.** Conidia; **l.** Germinating conidium. Scale Bars: c = 50 μm, d–l = 25 μm.

*Saprobic* on culms of bamboo. **Sexual morph**: Undetermined. **Asexual morph**: Hyphomycetous (Figs 1, 2). *Colonies* effuse, brown to dark-brown, hairy. *Mycelium* mostly immersed, composed of pale to dark brown, septate, branched, smooth, hyaline to subhyaline hyphae. *Conidiophores* macronematous, mononematous, septate, single or in groups of 2 or 3, erect, cylindrical, straight or slightly flexuous, olivaceous or brown, robust at the base 40–96 × 4–5.5 μm (x̅ = 69 × 5 μm, n = 10). *Conidiogenous cells* blastic, integrated, terminal, cylindrical, olivaceous or brown 9–19 × 4–5 μm (x̅ = 15 × 4.5 μm, n = 15). *Conidia* acrogenous, solitary, straight, obclavate, septate, thick-walled, rounded at the apex, truncate at the base, tapering towards apex, olivaceous or brown, 45–74 μm long (x̅ = 60.5 μm, n = 20), 5.5–9.5 μm at the widest (x̅ = 7.5 μm, n = 20).

Culture characteristics: Conidia germinated on PDA within 12 hours and germ tubes were produced from both ends. Colony reached 30 mm in 4 weeks at 26℃ on PDA media, circular, flat, surface rough, grey from above, brown from below, edge entire.

##### *Notes*

The morphological characteristics of *Distoseptispora bambusae* match well with the generic concept of *Distoseptispora* (Su et al. 2016). Multi-gene analyses showed that D. bambusae is a phylogenetically-distinct species, most closely related to D. suoluoensis, a species isolated from submerged wood in a freshwater habitat (Yang et al. 2018). *Distoseptispora bambusae* has shorter conidiophores (40–96 vs. 80–250 μm) and shorter conidia (45–74 vs. (65–) 80–125(–145) μm) than those of *D. suoluoensis* (Yang et al. 2018). Our two specimens of *D. bambusae* were similar in morphology, but polyblastic conidiogenous cells were observed from the Chinese specimen, while the Thai specimen has only monoblastic conidiogenous cells. These may be due to geographical differences and the different observation period. Although the two strains clustered together with short branches in the phylogenetic tree, comparisons of ITS sequences showed that there are 3 bp (base pair) differences without gaps between two strains and we identified them as the same species following the guidelines for species delineation proposed by Jeewon and Hyde (2016).

#### Etymology

Bambusae, referring to the host.

### Distoseptispora tectonae

Doilom & K.D. Hyde Fungal Diversity 81: 222 (2016)

72524FBE-F283-56B0-BE4E-649E0D85BC45

552223

#### Materials

- **Type status:** Other material. **Occurrence:** catalogNumber: MFLU 20–0262; **Taxon:** scientificName: Distoseptispora tectonae; class: Sordariomycetes; order: Distoseptisporales; family: Distoseptisporaceae; **Location:** country: Thailand; stateProvince: Chiangrai; locality: Mae Fah Luang University, Botanical Garden; verbatimElevation: 390 m

#### Description

**Figure 3.**
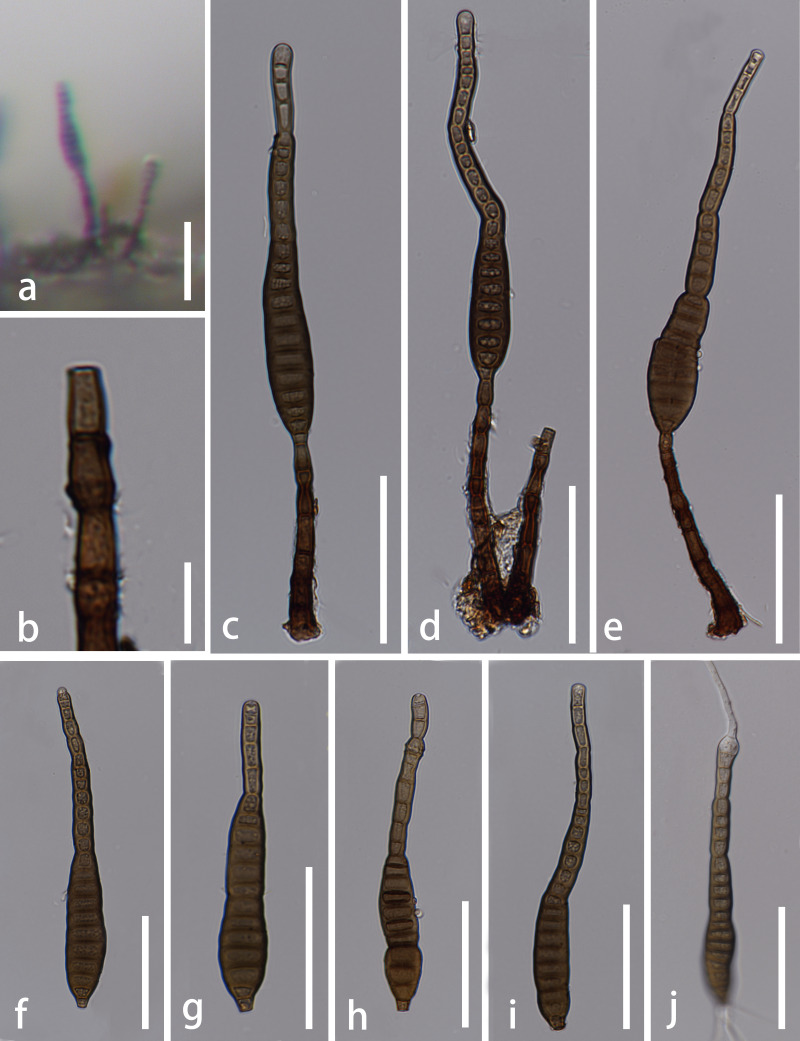
*Distoseptispora tectonae* (MFLU 20–0262). **a.** Colonies on natural substrate; **b.** Conidiogenous cell; **c**–**e.** Conidiophores and conidia; **f**–**i.** Conidia; **j.** Germinating conidium. Scale bars: b = 10 μm, a, c–j = 50 μm.

*Saprobic* on stems of dead wood. **Sexual morph**: Unknown. **Asexual morph**: Hyphomycetous (Fig. 3). *Colonies* effuse, brown to dark brown, hairy. *Mycelium* mostly immersed, composed of brown, septate, branched hyphae. *Conidiophores* macronematous, mononematous, septate, single or in groups of two, straight or slightly flexuous, cylindrical, dark brown, 34–95 × 5–8 μm (x̅ = 61.5 × 6 μm, n = 15). *Conidiogenous cells* integrated, terminal, monoblastic, cylindrical, brown. *Conidia* acrogenous, solitary, straight or slightly flexuous, rostrate, 11–23-distoseptate, differently constricted at the septa, thick-walled, truncate at the base, tapering towards apex, brown at the base, pale brown at the apex, 89–176 μm long (x̅ = 121 μm, n = 25), 12–19 μm at the widest (x̅ = 15 μm, n = 25).

*Culture characteristics*: Conidia germinated on PDA within 12 hours and germ tubes were produced from both ends. On PDA, colony circular, reaching 40 mm diam after 4 weeks at 26℃, brown from above, dark brown from below, surface flat and slightly rough, edge entire.

##### *Notes*

*Distoseptispora tectonae* was introduced by Hyde et al. (2016), from a terrestrial habitat in Thailand. *Distoseptispora tectonae* has macronematous, cylindrical, septate conidiophores, monoblastic, integrated, terminal, cylindrical conidiogenous cells and obclavate, straight or slightly curved, septate, smooth conidia. Our collection was also from Thailand. The morphological characters of our collection are the same as in the holotype, except that our isolate has longer and wider conidiophores (34–95 × 5–8 μm vs. up to 40 × 4–6 μm) and less septa (11–23 vs. 20–28), compared to those of *D. tectonae* MFLUCC 12–0291. In this study, we also provide new sequences for D. tectonae.

## Analysis

**Figure 4.**
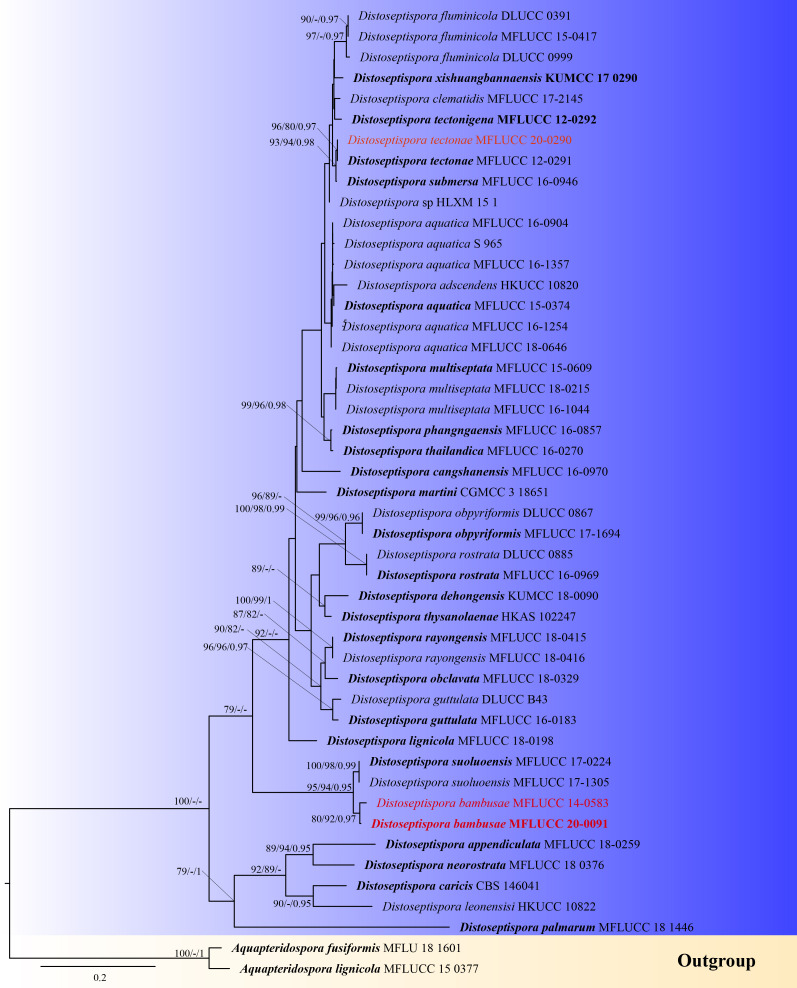
Maximum Likelihood (RAxML) tree, based on analysis of a combined dataset of LSU, ITS and *RPB2* sequence data. Bootstrap support values for ML and MP greater than 75% and Bayesian posterior probabilities greater than 0.95 are given near nodes, respectively. The tree is rooted with *Aquapteridospora fusiformis* (MFLU 18–1601) and *A. lignicola* (MFLUCC 15–0377). The ex-type strains are indicated in bold and the new isolates are in red.

Partial nucleotide sequences of the LSU, ITS and *RPB2* were used to determine the phylogenetic position of the taxa isolated. Sequences of 47 strains retrieved from GenBank, representing species of *Distoseptispora* and two outgroups *A. fusiformis* (MFLU 18–1601) and *A. lignicola* (MFLUCC 15–0377), were analysed. Single gene analyses were done to compare the topologies and clade stabilities, respectively. Nucleotide substitution models were selected by jModelTest2 on XEDE (Drummond et al. 2012). For the ITS and *RPB2* dataset, the GTR+I+G model was selected, for LSU, the TIM2+I+G. The manually-adjusted LSU, ITS and *RPB2* alignment comprised a total of 2,246 characters (768 for LSU; 436 for ITS; 1,042 for *RPB2*), including coded alignment gaps. Amongst them, 1,471 characters were constant, 195 variable characters were parsimony-uninformative and number of parsimony-informative characters was 580. One thousand equally most parsimonious trees (Tree length = 1799, CI = 0.640, RI = 0.733, RC = 0.469, HI = 0.360) were yielded from the heuristic search. MP, ML and Bayesian analyses of the combined dataset inferred similar topologies, respectively. The "most likelihood" tree is presented (Fig. 4).

In the phylogenetic analyses, generated by ML, MP and BI analysis, the two *Distoseptispora bambusae* isolates clustered with strong support (80%, 92%, 0.97). They formed a sister clade with *D. suoluoensis* with high support (95%, 94%, 0.95). Our isolate *D. tectonae* (MFLUCC 20–0090) grouped with *D. tectonae* (MFLUCC 12–0291) with strong ML, MP and BI support (96%, 80%, 0.97), indicating they are the same species.

## Discussion

In this study, two collections from China and Thailand, representing a new *Distoseptispora* species, is introduced, based on morphology and phylogenetic analysis. The two samples were both found on bamboo from terrestrial habitats. It is the fourth species found from medicinal plants. The other three are *D. palmarum*, *D. thailandica* and *D. xishuangbannaensis* (*Tibpromma et al. 2018, Hyde et al. 2019*).

*Distoseptispora* species does not seem to have specific habitat preferences. Most of them are reported from submerged wood in freshwater habitats, while some species have been introduced from terrestrial habitats (Luo et al. 2018, Tibpromma et al. 2018, Hyde et al. 2019, Luo et al. 2019, Phookamsak et al. 2019). So far, *Distoseptispora* were only found in China and Thailand. They may exist in other countries, waiting to be discovered on the basis of their diverse habitats.

The asexual morph of *Distoseptispora* is similar to *Sporidesmium* in producing holoblastic, euseptate or distoseptate conidia and blastic, terminal conidiogenous cells (Shenoy et al. 2006, Luo et al. 2018, Yang et al. 2018). Sexual morphs of *Distoseptispora* have not been reported.

*Acrodictys martini* was transferred to *Distoseptispora* as *D. martini* by Xia et al. (2017), based on their phylogenetic analysis. However, this species morphologically resembles *Acrodictys* rather than *Distoseptispora*. Therefore, the molecular data of *Distoseptispora martini* may need further verification (Luo et al. 2018).

It is interesting to note that, in most species of *Distoseptispora*, the conidia are longer than their conidiophores, while in some, they are shorter than their conidiophores. However, this characteristic does not reflect their phylogenetic position. For example, *D. obpyriformis* Z.L. Luo & H.Y. Su, a species that has long conidia and short conidiophores and *D. rostrata* Z.L. Luo, K.D. Hyde & H.Y. Su that has longer conidiophores, but shorter conidia, form a sister clade in the phylogenetic tree.

## Supplementary Material

XML Treatment for Distoseptispora bambusae

XML Treatment for Distoseptispora tectonae

## Figures and Tables

**Table 1.** Primers and PCR protocols.

Locus	Primer	PCR protocol	Reference
Internal Transcribed Spacer (ITS)	ITS5 ITS4	1. 94℃ – 3 min 2. 94℃ – 30 s 3. 52℃ – 30 s 4. 72℃ – 1 min 5. Repeat 2–4 for 35 cycles 6. 72℃ – 8 min 7. 4℃ on hold	White et al. (1990)
				Large Subunit rRNA (LSU, 28S)	LR0R LR5	Same protocol as ITS region	White et al. (1990), Rehner and Samuels (1995)
								RNA polymerase II Subunit 2 (*RPB2*)	*RPB2*-5f *RPB2*-7cR	1. 94℃ – 3 min 2. 94℃ – 20 sec 3. 55℃ – 30 sec 4. 72℃ – 1 min 5. Repeat 2–4 for 40 cycles 6. 72℃ – 10 min 7. 4℃ on hold	Liu et al. (1999)

**Table 2.** GenBank accession numbers of isolates included in this study. The newly-obtained strains are are indicated with ^※^ after collection number. Ex-type strains are in bold. **Abbreviation: CBS**: CBS-KNAW Fungal Biodiversity Centre, Utrecht, The Netherlands; **CGMCC**: China General Microbiological Culture Collection Center, Institute of Microbiology, Chinese Academy of Sciences, Beijing, China; **DLUCC**: Dali University Culture Collection, Yunnan, China; **HKUCC**: The University of Hong Kong Culture Collection, Hong Kong, China; **HKAS**: Kunming Institute of Botany Academia Sinica, Yunnan, China, **ICMP**: International Collection of Microorganisms from Plants, Auckland, New Zealand; **MFLU**: the herbarium of Mae Fah Luang University, Chiang Rai, Thailand; **MFLUCC**: Mae Fah Luang University Culture Collection, Chiang Rai, Thailand. Additional sequence of *D. bambusae* MFLUCC 20–0091: SSU: MT232716, TEF: MT232880

Species	Strain	ITS	LSU	*RPB2*
***Aquapteridospora lignicola***	**MFLUCC 15–0377**		**KU221018**	
***Aquapteridospora fusiformis***	**MFLU 18–1601**	**MK828652**	**MK849798**	
***D. aquatica***	**MFLUCC 15–0374**	**NR154040**	**KU376268**	
*D. aquatica*	S-965	MK828647	MK849792	MN124537
*D. aquatica*	MFLUCC 18–0646	MK828648	MK849793	
*D. aquatica*	MFLUCC 16–0904	MK828649	MK849794	
*D. aquatica*	MFLUCC 16–1254		MK849795	
*D. aquatica*	MFLUCC 16–1357	MK828650	MK849796	
***D. bambusae***	**MFLUCC 20–0091^※^**	MT232713	MT232718	MT232881
*D. bambusae*	MFLUCC 14–0583^※^	MT232712	MT232717	MT232882
***D. cangshanensis***	**MFLUCC 16–0970**	**MG979754**	**MG979761**	
***D. caricis***	**CBS 146041**	**MN562124**	**MN567632**	**MN556805**
***D. dehongensis***	**KUMCC 18–0090**	**MK085061**	**MK079662**	
*D. fluminicola*	MFLUCC 15–0417	NR154041	KU376270	
*D. fluminicola*	DLUCC 0391	MG979755	MG979762	
*D. fluminicola.*	DLUCC 0999	MG979756	MG979763	
***D. guttulata***	**MFLUCC 16–0183**	**MF077543**	**MF077554**	
*D. guttulata*	DLUCC B43	MN163011	MN163016	
*D. leonensisi*	HKUCC 10822		DQ408566	DQ435089
***D. lignicola***	**MFLUCC 18–0198**	**MK828651**	**MK849797**	
***D. martini***	**CGMCC 3.18651**	**KU999975**	**KX033566**	
*D. multiseptata*	MFLUCC 16–1044	MF077544	MF077555	MF135644
***D. multiseptata***	**MFLUCC 15–0609**	**KX710145**	**KX710140**	
*D. multiseptata*	MFLUCC 18–0215		MN163013	MN174864
***D neorostrata***	**MFLUCC 18–0376**	**MN163008**	**MN163017**	
***D. obclavata***	**MFLUCC 18–0329**	**MN163012**	**MN163010**	
***D. obpyriformis***	**MFLUCC 17–1694**		**MG979764**	**MG988415**
*D. obpyriformis*	DLUCC 0867	MG979757	MG979765	MG988416
***D. palmarum***	**MFLUCC 18–1446**	**MK085062**	**MK079663**	**MK087670**
***D. phangngaensis***	**MFLUCC 16–0857**	**MF077545**	**MF077556**	
***D. rostrata***	**MFLUCC 16–0969**	**MG979758**	**MG979766**	**MG988417**
*D. rostrata*	DLUCC 0885	MG979759	MG979767	
***D. submersa***	**MFLUCC 16–0946**	**MG979760**	**MG979768**	**MG988418**
*D. suoluoensis*	MFLUCC 17–1305	MF077547	MF077558	
***D. suoluoensis***	**MFLUCC 17–0224**	**MF077546**	**MF077557**	
*Distoseptispora*. sp	HLXM–15–1		KU376269	
***D. rayongensis***	**MFLUCC 18–0415**	**MH457172**	**MH457137**	**MH463255**
*D. rayongensis*	MFLUCC 18–0416	MH457173	MH457138	MH463256
***D. tectonae***	**MFLUCC 12–0291**	**KX751711**	**KX751713**	**KX751708**
*D. tectonae*	MFLUCC 20–0090^※^	MT232714	MT232719	
***D. tectonigena***	**MFLUCC 12–0292**	**KX751712**	**KX751714**	**KX751709**
***D. thailandica***	**MFLUCC 16–0270**	**MH275060**	**MH260292**	
***D. thysanolaenae***	**HKAS 102247**	**NR164041**	**MK064091**	
***D. xishuangbannaensis***	**KUMCC 17–0290**	**MH275061**	**MH260293**	**MH412754**
